# Use of an Extract of Indian Sacred Plant *Ocimum sanctum* as an Anticariogenic Agent: An *in vitro* Study

**DOI:** 10.5005/jp-journals-10005-1292

**Published:** 2015-08-11

**Authors:** Reshma Kochikar Pai, Sham S Bhat, Afreen Salman, Jagadish Chandra

**Affiliations:** Reader, Department of Pedodontics and Preventive Dentistry Yenepoya Dental College, Mangalore, Karnataka, India; Vice Principal, Professor and Head, Department of Pedodontics and Preventive Dentistry Yenepoya Dental College, Mangalore, Karnataka, India; Senior Lecturer, Department of Pedodontics and Preventive Dentistry Yenepoya Dental College, Mangalore, Karnataka, India; Professor, Department of Microbiology, Yenepoya Medical College Karnataka, India

**Keywords:** Antimicrobial activity, Chlorhexidine, Dental caries, Microorganisms, Ocimum sanctum.

## Abstract

**Aim:** To analyze the efficacy of three different concentrations of *Ocimum sanctum* extract against various microorganisms, *Streptococcus mutans, Streptococcus mitis, Streptococcus sanguis, Lactobacillus acidophilus.*

**Materials and methods:** Ethanolic extract of *Ocimum sanctum* was prepared by the hot extraction method. The extract was diluted with an inert solvent, dimethyl sulfoxide to obtain 3 different concentrations (2.5, 5 and 10%) of the extract. 0.2% chlorhexidine was used as a positive control and dimethyl sul-foxide was used as a negative control. The extract, along with the controls, was then subjected to microbiological investigation to determine which concentration among the 3 different concentrations of extract gave a wider inhibition zone against *S. mutans, S. mitis, S. sanguis, L. acidophilus.* The zones of inhibition were measured in millimeters.

**Results:** *Ocimum sanctum* leaf extract demonstrated maximum antimicrobial activity against microorganisms responsible for dental caries at the 10% concentration level although 5 and 2.5% were also effective. Maximum activity was seen against S. *mutans* and S. *sanguis* with 10% extract.

**Conclusion:** *Ocimum sanctum* leaf extract was effective against all the microorganisms.

**How to cite this article:** Pai RK, Bhat SS, Salman A, Chandra J. Use of an Extract of Indian Sacred Plant *Ocimum Sanctum* as an Anticariogenic Agent: An *in vitro* Study. Int J Clin Pediatr Dent 2015;8(2):99-101.

## INTRODUCTION

Tulsi known in English as Holy Basil and botanically called *Ocimum sanctum* is described as a sacred and medicinal plant in Ancient literature. Tulsi is also called as the ‘Elixir of Life’ since it promotes the longevity. It is an important symbol of Hindu religious tradition and is found in most of the Indian homes and worshipped.^1-3^

The name Tulsi which in Sanskrit means ‘The incomparable One’, has got two varieties, Krishna Tulsi (black) and Rama Tulsi (green).^4,5^ Their chemical constituents are similar but Krishna Tulsi possesses great medicinal value as mentioned in Charaka Samhita, an ancient Indian literature. It is a herb that is bestowed with enormous antimicrobial substances and used to treat a variety of illnesses ranging from diabetes, arthritis, bronchitis, skin diseases, etc.^4,5^

Dental caries is one of the most common microbial disease affecting humans particularly children. The main bacteria involved in caries are *Streptococcus mutans, Streptococcus mitis, Streptococcus sanguis* and *Lactobacillus acidophilus.*

The present management of caries involves tooth brushes, mouth rinses, toothpastes, gels, etc.^6^ The incorporation of broad spectrum antimicrobial mouth rinses as adjuncts to patient’s oral hygiene regimens has assumed greater importance with the recognition that most children are unable to maintain adequate oral hygiene.^7^

Many of the currently available mouth rinses have drawbacks, such as alteration of taste, burning sensation, staining of teeth, etc. This has necessitated the search for alternative mouth rinsing agents especially in children. The herbal preparations are considered moderate in efficacy and are less toxic than the most commonly used pharmaceutical mouth rinses.^8^

As studies related to herbal mouth rinses are lacking, research in this area is necessary to generate the required evidence. Hence in our study, we have selected *O. sanctum* (Krishna Tulsi) and analyzed the efficacy of three different concentrations of its extract against various microorganisms responsible for caries.

## MATERIALS AND METHODS

### Collection of Suitable Plant Material

*Ocimum sanctum* was collected from courtyards. The plant was authenticated by Dr Krishnakumar G, Chairman, Department of Applied Botany, Mangalore University. Leaves suitable for extraction were plucked and were washed under running tap water followed by sterilized distilled water. The 200 gm leaves were dried in sunlight and powdered.

### Preparation of *Ocimum Sanctum* Ethanolic Extract

*Ocimum sanctum ethanolic extract (OSEE):* A weighed quantity (30 gm) of the coarse powder was extracted with ethanol (90%) in a Soxhlet apparatus. The extract was concentrated on a water bath at a temperature not exceeding 60° C. The percentage yield of the extract was 10%.^9,10^

### Preparation of different Concentrations of *Ocimum sanctum* Extract

One gm of the extract was dissolved in 10 ml of dimethyl sulfoxide to obtain 10% concentration of the extract. From this 5 and 2.5% dilutions were prepared in sterile distilled water. 0.2% chlorhexidine was used as a positive control and dimethyl sulfoxide was used as a negative control.

## MICROBIOLOGICAL ANALYSIS

### Standard Strains and Culture Conditions

Pure strains of *S. mitis* ATCC 6249, *S. mutans* ATCC 25175 *S. sanguis* ATCC 10556, *L. acidophilus* ATCC 4358 were purchased from Himedia.

### Culture Media used for Testing Antimicrobial Activity of *Ocimum Sanctum* Leaf Extract

*Streptococcus* species were cultured in Brain heart infusion agar (Himedia) and *L. acidophilus* in *Lactobacillus* MRS Agar (Himedia).

Lyophilized preparations of standard strains of above mentioned bacteria were inoculated into combination of 1 ml of Brain heart infusion broth and 1 ml of Thioglycollate broth and incubated at 37°C in Anaerobic chamber for 48 hours. Subcultures were made on Brain heart infusion agar and *Lactobacillus* MRS Agar respectively for Streptococci and *Lactobacilli.*

Standard inoculums were prepared for each bacterium to match McFarland’ turbidity standard tube 0.5. From the inoculums Lawn culture were made on Brain heart Infusion agar and *Lactobacillus* MRS Agar. 8 mm diameter wells were cut in the media. Using micropipette 25 micro liters of various concentrations (10, 5 and 2.5%) of *O. sanctum* extract and undiluted DMSO and 0.2% chlorhexidine where transferred into these wells. All the plates were incubated in anaerobic chamber for 48 hours at 37°C. After removing the plates from anaerobic chamber, diameter of zone of inhibition around the wells were measured.^11^

**Fig. 1:**
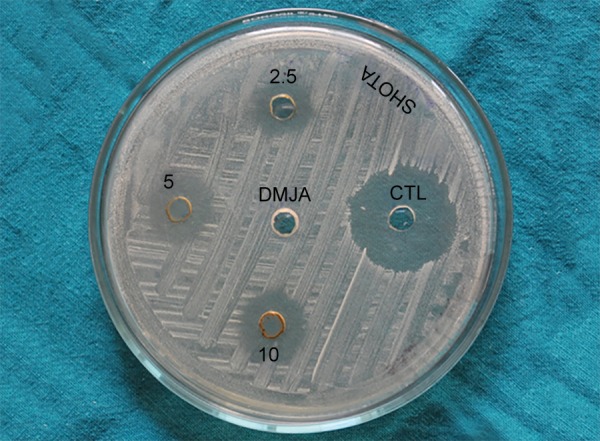
Zone of inhibition for S. *mutans*

**Table 1:** **Table 1:** Zone of inhibition of microorganisms at different concentrations

*Organism name*		*Dimethyl sulfoxide*		*10% Ocimum sanctum extract*		*5% Ocimum sanctum extract*		*2.5% Ocimum sanctum extract*		*Chlorhexidine 0.2 %*	
*Streptococcus mutans*		0 mm		22 mm		18 mm		12 mm		28 mm	
*Streptococcus sanguis*		0 mm		20 mm		16 mm		12 mm		23 mm	
*Streptococcus mitis*		0 mm		17 mm		12 mm		10 mm		22 mm	
*Lactobacillus acidophilus*		0 mm		17 mm		15 mm		14 mm		24 mm	

## RESULTS

The data obtained were appraised observationally. All the bacterial strains were resistant to dimethyl sulfoxide. For 0.2 % chlorhexidine (positive control) a wider zone of inhibition of 28 mm was achieved for *S. mutans* (Fig. 1), followed by 24, 23 and 22 mm for *L. acidophilus, S. sanguis* and *S. mitis* respectively (Table 1). Among the different concentrations of *O. sanctum* extract used, 10% extract showed a wider zone when compared to 5% and 2.5 % extract. Maximum activity was seen against *S. mutans* and *S. sanguis* with 10% extract.

## DISCUSSION

There is widespread emergence of resistance among microbial pathogens against currently available antimicrobials. Traditional plants have been proved to be better source in the search for novel antimicrobial compounds. Among the plants known for the medicinal value tulsi is safe, economical, effective and easily available.

Eugenol (1-hydroxy - 2 - methoxy-4 - allyl benzene), the active constituent present in *O. sanctum,* is largely responsible for the therapeutic potential of tulsi. The other important constituents include ursolic acid and carvacrol. The antimicrobial activity of *O. sanctum* can be attributed to these constituents.^12^ Hence, this study was designed to find the efficacy of *O. sanctum* extract as an antimicrobial agent.

In this study, we have used the leaves of black tulsi (Krishna tulsi). Ethanol was used as a solvent because the essential oils in *O. sanctum* are more soluble in alcohol when compared to distilled water. Dimethyl sulfoxide, an inert solvent was used to dilute the extract to neutralize the effect of alcohol which itself is an antiseptic, attributing the results solely to tulsi.

Agarwal et al determined the antimicrobial activity of *O. sanctum* against *S. mutans* and found that 4% concentration of *O. sanctum* extract showed the widest zone of inhibition against *S. mutans.^13^*

The effect of 0.2% chlorhexidine mouth rinse, Listerine mouth rinse and 4% *O. sanctum* tulsi extract mouth rinse on salivary *S. mutans* level were compared by Agarwal et al, it was found that and *O. sanctum* extract was as effective as chlorhexidine and Listerine in reducing the salivary *S. mutans* level.^14^

In our study, 10% *O. sanctum* leaf extract showed wide zone of inhibition against all the microorganisms― *S. mutans, S. sanguis, S. mitis* and *L. acidophilus.* Five percent and 2.5% concentrations were also effective in inhibiting the microbial activity although it was not as high as 10% concentration. Chlorhexidine was found to be more effective compared to *O. sanctum* leaf extract. But the well known side effects of chlorhexidine like staining of the teeth and restoration, alteration of taste sensation and development of resistant microorganisms may limit the long-term use of chlorhexidine. In comparison with herbal medicines, tulsi is abundantly available, easily accessible, economically feasible and culturally acceptable and may possess minimal side effects, hence it can be recommended for long-term use.

According to current knowledge, this is the first study which determines the antimicrobial activity of *O. sanctum* against four different microorganisms causing dental caries. Although 10% extract of *O. sanctum* showed maximum antimicrobial potential, this needs to be confirmed with further long-term studies to investigate the effect of *O. sanctum* on dental caries before it can be confidently recommended. Also *in vivo* studies may be needed for clinical efficacy.

## CONCLUSION

*Ocimum sanctum* leaf extract demonstrated antimicrobial activity against microorganisms responsible for dental caries at the 10% concentration level although 5 and 2.5% were also effective. This is an encouraging result which may favor the promotion of *O. sanctum* as a mouth rinse.

## References

[1] Kumar PK, Kumar MR, Kavita K, Singh J, Khan R (2012). Pharmacological actions of ocimum sanctum–a review article.. IJAPBC.

[2] Sanctum LDO (2010). (tulsi): A potent medicinal herb.. Webmed Cental Pharmacol.

[3] Pandey G, Madhuri S (2010). Pharmacological activities of ocimum sanctum (tulsi): a review.. Int J Pharmaceutical Sci Review and Res.

[4] Mondal S, Miranda BRRB, Sushil CM (2009). The science behind sacredness of Tulsi (Ocimum sanctum LINN.).. Ind J of Physiol Pharmacol.

[5] Goyal P, Kaushik P (2011). In vitro evaluation of antibacterial activity of various crude leaf extracts of Indian Sacred Plant, Ocimum sanctum L.. British Microbiol Res J.

[6] Balakrishnan M, Simmonds RS, Tagg JR (2000). Dental caries is a preventable infectious disease.. Aust Dent J.

[7] Mandal ID (1998). Chemotherapeutic agents for controlling plaque and gingivitis.. J Clin Periodont.

[8] Lewis ME (2001). Should we be concerned about herbal remedies?. J Ethnopharmacol.

[9] Abhimanyu KJ, Meenakski J, Jagdeep K (2012). Ethanolic extract of Ocimum sanctum Azadirachta Indica and Withania Somnif-era cause apoptosis in sitla cells.. RJPBCS.

[10] Mishra P, Mishra S (2011). Study of antibacterial activity of Ocimum Sanctum extract against gram positive and gram negative bacteria.. Am J Food Technol.

[11] Oboh IE, Akerele JO, Obasuyi . (2007). Antimicrobial activity of ethanol extract of aeril parts of sida acuta burm.. Tropical J Phamaceut Res.

[12] Prakash P, Gupta N (2005). Therapeutic uses of ocimum sanctum Linn (tulsi) with a note on eugenol and its pharmacological actions–a short review.. Indian J Physiol Pharmacol.

[13] Agarwal P, Nagesh L, Krishna M (2010). Evaluation of antimicrobial activity of various concentration of tulsi ( Ocimum Sanctum) extract against Streptococcus mutans: an in vitro study.. Indian J Dent Res.

[14] Agarwal P, Nagesh L (2011). Comparative evaluation of efficacy of 0.2% chlorhexidine, Listerine and tulsi extract mouth rinses on salivary streptococcus mutans count of high school chil-dren-RCT.. Contemporary Clinical Trials.

